# Comparative Analysis of Predicted Plastid-Targeted Proteomes of Sequenced Higher Plant Genomes

**DOI:** 10.1371/journal.pone.0112870

**Published:** 2014-11-13

**Authors:** Scott Schaeffer, Artemus Harper, Rajani Raja, Pankaj Jaiswal, Amit Dhingra

**Affiliations:** 1 Department of Horticulture, Washington State University, Pullman, WA, United States of America; 2 Molecular Plant Science Graduate Program, Washington State University, Pullman, WA, United States of America; 3 2082 Cordley Hall, Department of Botany and Plant Pathology, Oregon State University, Corvallis, OR, United States of America; University of California – Davis, United States of America

## Abstract

Plastids are actively involved in numerous plant processes critical to growth, development and adaptation. They play a primary role in photosynthesis, pigment and monoterpene synthesis, gravity sensing, starch and fatty acid synthesis, as well as oil, and protein storage. We applied two complementary methods to analyze the recently published apple genome (Malus × domestica) to identify putative plastid-targeted proteins, the first using TargetP and the second using a custom workflow utilizing a set of predictive programs. Apple shares roughly 40% of its 10,492 putative plastid-targeted proteins with that of the Arabidopsis (Arabidopsis thaliana) plastid-targeted proteome as identified by the Chloroplast 2010 project and ∼57% of its entire proteome with Arabidopsis. This suggests that the plastid-targeted proteomes between apple and Arabidopsis are different, and interestingly alludes to the presence of differential targeting of homologs between the two species. Co-expression analysis of 2,224 genes encoding putative plastid-targeted apple proteins suggests that they play a role in plant developmental and intermediary metabolism. Further, an inter-specific comparison of Arabidopsis, Prunus persica (Peach), Malus × domestica (Apple), Populus trichocarpa (Black cottonwood), Fragaria vesca (Woodland Strawberry), Solanum lycopersicum (Tomato) and Vitis vinifera (Grapevine) also identified a large number of novel species-specific plastid-targeted proteins. This analysis also revealed the presence of alternatively targeted homologs across species. Two separate analyses revealed that a small subset of proteins, one representing 289 protein clusters and the other 737 unique protein sequences, are conserved between seven plastid-targeted angiosperm proteomes. Majority of the novel proteins were annotated to play roles in stress response, transport, catabolic processes, and cellular component organization. Our results suggest that the current state of knowledge regarding plastid biology, preferentially based on model systems is deficient. New plant genomes are expected to enable the identification of potentially new plastid-targeted proteins that will aid in studying novel roles of plastids.

## Introduction

The plastid is an intracellular organelle derived from an endosymbiotic event wherein a free-living autotrophic photosynthetic bacterium was phagocytized by a separate heterotrophic organism [1]. These organelles have since become essential to plant survival and have been documented to participate in numerous biological processes including photosynthesis, storage of oils, and proteins, pigment synthesis and storage, monoterpene synthesis [2], gravity sensing [3], and starch and fatty acid synthesis [4]. Over an extensive period of evolution, large parts of the plastid genome are hypothesized to have integrated into the nuclear genome [5]. In higher plants, the vast majority of proteins constituting the plastid proteome are encoded by genes physically resident in the nuclear genome, with about 120 genes retained in the plastid genome, a number which varies between species [6]. Comparative genomic analysis between Arabidopsis (*Arabidopsis thaliana*) and cyanobacteria indicates that 18% of the Arabidopsis protein–coding genes were derived from events involving transfer of genetic material from the plastid to the nucleus [7]. In part, exchange of genetic material and related biological functionality has necessitated an orchestration of processes between the plastid and nucleus where the nucleus actively exerts control on all aspects of plastid function.

Plant cells have developed intricate mechanisms to import nuclear-encoded proteins to or across the three plastid membranes (outer, inner plastid envelope, thylakoid). The presence of multiple protein transport pathways have been shown to play a role in aiding protein transport across the inner and outer plastid envelopes; however, the vast majority of the plastid proteome is transported via the tic/toc pathway [8]. In order to utilize this pathway, most proteins possess a signal peptide which interacts with chaperones and is later cleaved. Stromal-targeting peptide sequences, while not conserved, possess some similarities in amino acid composition. These targeting peptides are typically comprised of a relatively high abundance of serine and threonine residues [9], [10] and are positively charged [11]. There are also some proteins that do not have any canonical signaling peptides and yet localize to plastids [12], [13], [14]. Therefore, the signaling prediction programs provide a good reference point to initiate an understanding of the plastid-targeted proteome for any new species, but as predictions, they do require experimental validation.

Due largely to the technical complexity with whole plastid proteome characterization, transcriptome or genome sequences have become a widely used dataset to predict plastid-targeted motifs. Such an approach also enables the identification of plastid-targeting proteins in a spatial and temporal context. Prediction of subcellular localization has been reported to be performed with software such as PCLR [15], iPSORT [16], TargetP [17], [18], and PREDOTAR [19] amongst many other programs. Most prediction methods exploit the presence of an N-terminal signal sequence to predict cellular localization. Of these, TargetP was recommended to be most successful in prediction and was comparable to PCLR, with each having sensitivity values of 0.72 [20].

The Rosaceae family represents a unique diversity in fruit development, which is unrealized in the many model plants whose genomes have been sequenced [21], [22], [23], [24], [25], [26]. Pomes (apples and pears), stone fruits (cherry and peach), and aggregate fruits (strawberry and raspberry) display a diversity that suggests the presence of novel metabolic processes, and is supported by a large number of genes which far exceeds the number of genes in Arabidopsis. While fruit development in these Rosaceae species differs vastly, the ubiquitous process of the plastidial transition from a chloroplast to chromoplast is often assumed to be conserved. Within Rosaceae fruit, plastids play extremely important roles in determining fruit quality and organoleptic appeal as they are the site for synthesis of carotenoids [27], [28], monoterpenes [2], fatty acids [4] and aromatic amino acids. Many of these compounds have been linked to human health and nutrition [29], [30]. Plastids are also important in converting starch into various types of carbohydrate and sugars in developing fruits [31].

The plastid structure has also been reported to differ between different tissues of the fruit. Phan [32] reported the presence of a large single granum comprised only of stacked thylakoid membranes in the plastids of the endocarp tissue of apple. In addition, chloroplasts with leaf-like thylakoid and grana organization in the outer six cell layers of mature apple fruit and presence of globular chromoplasts in epidermal cells were described. It is expected that differences in structure, physiology and biochemistry in the Rosaceae fruit plastids as well as other non-model systems will assist in identifying novel processes associated with plastids in plants.

In this study we tested the three primary hypotheses using a bioinformatics approach, (1) The total number and composition of the plastid-targeted protein coding genes in apple, a model representative of Rosaceae, that is taxonomically different from Arabidopsis, (2) The plastid-targeted protein coding genes are under transcriptional control during apple fruit development and (3) There is a subset of unique plastid-targeted protein coding genes that are unique and novel to each plant species.

In order to test the first hypothesis, we performed an in-depth computational analysis predicting the plastid-targeted proteome of apple and compared it with Arabidopsis resulting in the identification of a much larger number of plastid-targeted genes with nearly 4000 plastid-targeted protein coding genes being unique to apple. The second hypothesis was tested by reanalyzing publically available apple transcriptome data which revealed the presence of co-expression profiles of plastid-targeted genes and their association to development and metabolism. Finally, the third hypothesis was tested by extending the custom analysis workflow to an inter-genera comparison between six published genomes: *Arabidopsis thaliana*, *Vitis vinifera*, *Prunus persica*, *Populus trichocarpa*, *Fragaria vesca*, and *Solanum lycopersicum* resulting in the identification of plastid-targeted proteins unique to each species. A core set of 737 *Arabidopsis thaliana* proteins, highly enriched in photosynthesis and primary metabolism gene ontology (GO) terms, were identified to have homologous plastid-targeted proteins in all investigated species.

## Materials and Methods

### TargetP-based prediction of *Malus* × *domestica* plastid proteome

The *Malus × domestica* predicted protein set was obtained from the apple genome sequencing project [26]. Protein sequences were analyzed using TargetP using plant networks with default parameters [17], [18]. All sequences with predicted chloroplast transit peptides were compiled into a new dataset and were sorted based on length using USEARCH [33].

### Custom protein targeting analysis

A part of the functional annotation pipeline was applied to identify organelle ‘plastid’ targeted gene products encoded by the apple genome [26]. The peptide sequences were analyzed first through InterProScan [34] results provided by the genome consortium [26], followed by in-house analysis using the SignalP [17], Predotar [19] and TMHMM [35]. InterPro provided the domain annotations, and any genes/peptides with transposable element/domain annotations were filtered out for further analysis. The next steps of the pipeline employed: (1) SignalP to predict localization to the mitochondrial or plastid or secretion pathway, plus providing signal peptide cleavage sites, (2) Predotar to predict localization to either or both the mitochondrion or plastid, and (3) TMHMM to identify predicted transmembrane domains in the protein sequences. After collecting these annotations, standardized protocols for assigning the annotations were adopted [24]. The higher quality scores with reviewed after computational analyses (RCA) were selected if the scores of 0.75 and greater were predicted for TargetP and Predotar and two or more transmembrane annotations were predicted by the TMHMM. The parameters selected for inferred by electronic annotation (IEA) include the scores of 0.5–0.749 for TargetP and Predotar and one/single transmembrane domain suggested by the TMHMM. The majority of these annotations were IEA evidence codes. If the annotations overlapped for gene products that had plastid-targeting predicted from TargetP and Predotar and membrane spanning domains identified by the TMHMM, then the suggested location of the targeted protein was ‘plastid membrane’.

The Inparanoid algorithm [36] was used to find orthologous genes and paralogous genes that arise by duplication events. The pipeline was discussed in the *Fragaria vesca* genome paper [24]. For this study, the analysis included the peptide sequences from 22 species, including *Arabidopsis thaliana, Brachypodium distachyon, Caenorhabitis elegans, Chlamydomonas reinhardtii, Danio rerio, Eschericia coli, Fragaria vesca, Glycine max, Homo sapiens sapiens, Zea mays, Malus × domestica, Mus musculus, Neurospora crassa, Oryza sativa, Physcomitrella patens, Populus trichocarpa, Saccharomyces cerevisiae and pombe, Selaginella moellendorffii, Sorghum bicolor, Synechosystis, and Vitis vinifera* to cover the tree of life with emphasis on fully/nearly complete and published genomes. The peptide sequences were downloaded from Phytozome.net for grapevine, *Selaginella, Physcomitrella, Chlamydomonas, Glycine, Populus, and Malus* from the genome portal [26], and *Gramene* [37] for rice, sorghum, maize and Arabidopsis. The remaining sequences were downloaded from Ensembl [38], [39].

### Identification of sequences unique to apple datasets compared to Arabidopsis

The *Arabidopsis thaliana* plastid-targeted gene set was obtained from the Chloroplast 2010 project website (www.plastid.msu.edu) [40]. Arabidopsis embryo lethal mutants were analyzed using TargetP [17], [18] and any chloroplast targeted proteins were added to the aforementioned dataset, as these were omitted from the Chloroplast 2010 database. Proteins predicted to target the apple plastid were then compared to plastid-targeted proteins from *Arabidopsis thaliana* using USEARCH [33]. Predicted plastid-targeted proteins were compared using two conditions: first, a global USEARCH was performed using 40% amino acid identity and 40% coverage (40/40), and a second global comparison was performed using 50% amino acid identity with 50% coverage (50/50). Header files of proteins unique to the *M. × domestica* dataset were compared between the TargetP-based method as well as the custom analysis to investigate any bias associated with either respective prediction technique.

### USEARCH-based multispecies comparative analysis of predicted plastid-targeted proteomes

Predicted coding sequences were collected from the genomes of *Fragaria vesca* (Woodland Strawberry) [24], *Vitis vinifera* (Grapevine) [22], *Solanum lycopersicum* ITAG1 release (Tomato) [23], *Prunus persica* (Peach) [21] and *Populus trichocarpa* (Black Cottonwood) [25]. Protein sequences were analyzed with TargetP [17], [18] using default parameters to predict localization. Sequences predicted to be plastid-targeted were organized into new files for each species. Comparisons were performed for each plastid-targeted dataset using USEARCH [33] 40/40 and 50/50 global parameters against the *Arabidopsis thaliana* plastid-targeted dataset, the entire Arabidopsis TAIR V10 protein set (Arabidopsis.org) [41], the predicted proteins from *Solanum lycopersicum*, as well as a file comprised of the sequences of the predicted plastid-targeted protein sequences of the other six species. All datasets were first sorted by length using USEARCH.

Further analysis was performed with USEARCH to identify those proteins present in the predicted plastid proteomes of all investigated species. To perform this analysis, the *Arabidopsis thaliana* putative plastid-targeted protein set was compared using USEARCH 40/40 global parameters separately against the plastid-targeted proteins from woodland strawberry, grapevine, tomato, peach, black cottonwood, and apple. Output files were then analyzed to identify those Arabidopsis sequences which had a match in the plastid-targeted proteomes of all species.

### UCLUST-based multispecies comparative analysis of predicted plastid-targeted proteomes

A second comparative analysis was performed using the clustering feature of the USEARCH package, UCLUST. In this analysis, the plastid-targeted protein sequences from the seven examined species were compiled into a single file and sorted by length. UCLUST was performed at 50% identity. The output was parsed to identify protein clusters with members from all seven species, as well as those clusters containing sequences from only one species.

### Determination of Jaccard's similarity coefficients

Two separate techniques were used to create the similarity matrices based upon Jaccard's coefficients. To calculate the value of an individual cell (the distance between species A and species B) we first determined if two genes were considered homologous. If a gene from the species A matches with another gene in species B, then both A and B are included in the intersection set. The result in the cell is the count of the intersection set divided by the sum of all the genes in both species. For the USEARCH-based approach, two genes match if they align to each other using 40/40 parameters. Alternatively, in the UCLUST-based approach two genes match if they belong to the same cluster.

### Blast2GO Gene Ontology analysis and GO term enrichment analysis

Sequences for all genes encoding unique or shared plastid-targeted proteins in the investigated apple, Arabidopsis, grapevine, peach, strawberry, black cottonwood, and tomato datasets were analyzed via Blast2GO [42], [43]. BLASTP was performed using the NCBI nr database with Blast2GO default parameters. Gene ontology mapping and annotation were also performed using default parameters with the August 2012 database. Following GO annotation, an Interpro scan [34], [44]was performed and results were merged with the GO annotations. Annotation augmentation was performed using ANNEX [45], followed by GO-slim with the goslim_plant.obo database. Kyoto Encyclopedia of Genes and Genomes (KEGG) information was downloaded from the KEGG Pathway Database [46], [47]. Datasets comprising those unique to each plastid-targeted proteome, as well as those shared between all seven species were investigated using Single Enrichment Analysis with agriGO [48] to identify enriched GO terms. Analysis was performed using the Fisher test for significance and adjusted using the Yekutieli multi test adjustment with the minimum mapping entries set to three. A significance level was set at 0.01 and all terms GO-terms with p-values lower than this cutoff were reported as enriched.

### Analysis of apple fruit gene expression

In order to ascertain if genes encoding plastid-targeted proteins in apple are expressed in fruits, as well as to identify co-expressed gene sets, microarray data from a previously published experiment were used [49]. The Janssen study measured the relative expression of about 13,000 features designed from apple fruit expressed sequence tags (ESTs) at 8 time points ranging from 0 days after anthesis (DAA) to 146 DAA. All EST sequences utilized in the microarray experiment were retrieved from NCBI and a BLASTX was performed against the predicted apple protein set generated from the apple genome [26]. The EST expression data were then assigned to the top protein hit. Sequences which were previously found to be plastid-targeted were extracted and their respective expression data were analyzed by determining relative expression to the lowest measured mean expression value. The Log2 of relative expression data were imported into and analyzed with MultiExperiment Viewer [50], [51]. Sequences were clustered using Cluster Affinity Search Technique [52] using Pearson Correlation and a threshold of 0.8. Blast2GO [42] was used to assign annotation to those proteins with associated gene expression data. Single Enrichment Analysis was performed with AgriGO [48] as previously described, however a chi-square test was used instead to determine statistical significance.

## Results

### Predicted Plastid-targeted Proteomes of *Malus* × *domestica*

**Figure 1:**
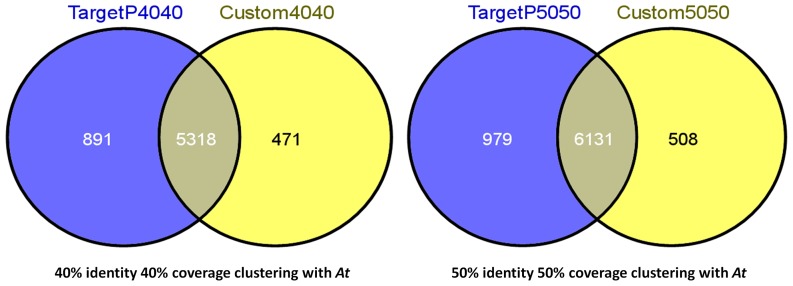
Venn diagrams displaying the predicted plastid-targeting proteins unique to apple compared to *Arabidopsis*. Two plastid-targeting methods, TargetP and a custom analysis method, were used to predict genes encoding plastid localized proteins. Sequences in these data sets were compared to *Arabidopsis* plastid-targeted proteins from the Chloroplast2010 project using USEARCH. Genes not clustered to *Arabidopsis* were compared between prediction methods displaying a high agreement between the methods. Venn diagrams were constructed using Venny (Oliveros, 2007).

The apple genome has a total of 57,386 predicted genes [26] nearly 30,000 more genes than Arabidopsis [41], [53]. We analyzed the complete apple gene set for cellular localization using two approaches, namely TargetP [17], [18] and a custom prediction method (see materials and methods section for details). TargetP predicted the presence of 10,492 plastid-targeted proteins in the apple genome, while the custom gene ontology-based analysis predicted 9,882 genes, with an overlap of 9,256. Each data set was then clustered with the Arabidopsis plastid-targeted protein set using USEARCH [33] with 40% identity and 40% coverage (40/40 parameters) to identify homologous protein sequences. The TargetP method and custom analysis predicted 6,209 and 5,789 plastid-targeted proteins respectively to be unique to the apple dataset. The two methods agreed upon 5,318 proteins (86% and 92% respectively) uniquely targeted to apple chloroplasts and absent from those of *Arabidopsis thaliana* (Figure 1). Alternative clustering using 50% identity and 50% coverage (50/50 parameters) resulted in less clustering with *Arabidopsis* sequences and, consequently, increased the number of proteins predicted to be unique to apple. Using these parameters 7,110 sequences were predicted to be unique to the apple plastid proteome by TargetP, 6,639 with the custom analysis, and a set of 6,131 agreed upon by the two methods.

In order to identify prediction biases between the custom analysis and TargetP, an agriGO [48] GO term enrichment was performed on the proteins predicted to be differentially targeted. No significant GO terms were found to be enriched in the 1,236 proteins predicted to target the plastid with TargetP. However, agriGO identified the GO terms oxygen binding (GO:0019825, p-value 3.2e-05), hydrolase activity (GO:0016787 p-value 6e-05) and catalytic activity (GO:0003824 p-value 3.3e-04) are enriched in the plastid-targeted-proteins unique to the custom analysis.

### Expression analysis of genes encoding plastid-targeted proteins in *Malus* × *domestica*

In order to test the hypothesis that plastid-targeted protein coding genes are under transcriptional control as the apple fruit develops, we reanalyzed data from a previously published microarray-based analysis of developing apple fruit [49]. Of the 13,000 unigene microarray probe sets studied, 2,698 were determined to map back to putative plastid-targeted proteins identified in this study, and represent a total of 2,224 unique sequences. Clustering of expression data using MultiExperiment Viewer [50], [51] identified 92 different expression clusters, however, only 64 of these had 5 or more members. Over 50% of the genes fit into 9 co-expression clusters. These co-expressed genes were annotated using Blast2GO to infer their functions. Expression data for each cluster are provided in File S1 as well as their associated GO term information (File S2).

Of the 11 main clusters investigated, only the two most populous clusters of co-expressed genes contained GO terms which were determined to be significantly enriched by agriGO analysis (Table 1). Cluster 1 had a single enriched biological process GO term of photosynthesis (GO:0015979) along with 12 enriched cellular component GO terms with thylakoid (GO:0009579) having the lowest p-value. Cluster 2 was enriched in the biological process GO terms lipid metabolic process (GO:0006629), secondary metabolic process (GO:0019748), biosynthetic process (GO:0009058), catabolic process (GO:0009056), transport (GO:006810), establishment of localization (GO:0051234), and localization (GO:0051179). Additionally, one molecular function GO term was enriched in cluster 2, catalytic activity (GO:0003824), along with 11 cellular component GO terms.

**Table 1:** Enriched GO terms associated with co-expressed genes encoding plastid-targeted proteins in developing apple.

	GO term	Ontology	Description	p-value	FDR
**Cluster 1**	GO:0015979	P	photosynthesis	7.80E-08	2.60E-05
	GO:0009579	C	thylakoid	5.50E-16	9.10E-14
	GO:0009536	C	plastid	4.70E-06	3.90E-04
	GO:0016020	C	membrane	1.10E-05	6.20E-04
	GO:0043231	C	intracellular membrane-bounded organelle	3.20E-05	1.10E-03
	GO:0043227	C	membrane-bounded organelle	3.20E-05	1.10E-03
	GO:0043229	C	intracellular organelle	6.90E-05	1.60E-03
	GO:0043226	C	organelle	6.90E-05	1.60E-03
	GO:0005623	C	cell	8.30E-05	1.70E-03
	GO:0044464	C	cell part	1.30E-04	2.30E-03
	GO:0044424	C	intracellular part	6.50E-04	1.10E-02
	GO:0044444	C	cytoplasmic part	7.00E-04	1.10E-02
	GO:0005622	C	intracellular	1.60E-03	2.20E-02
**Cluster 2**	GO:0006629	P	lipid metabolic process	8.40E-10	2.60E-07
	GO:0019748	P	secondary metabolic process	8.70E-05	1.30E-02
	GO:0009058	P	biosynthetic process	1.60E-04	1.60E-02
	GO:0009056	P	catabolic process	4.40E-04	3.40E-02
	GO:0006810	P	transport	9.20E-04	4.00E-02
	GO:0051234	P	establishment of localization	9.20E-04	4.00E-02
	GO:0051179	P	localization	9.20E-04	4.00E-02
	GO:0003824	F	catalytic activity	5.60E-04	3.80E-02
	GO:0005737	C	cytoplasm	7.00E-12	1.40E-09
	GO:0044464	C	cell part	1.80E-11	1.80E-09
	GO:0005623	C	cell	4.90E-11	3.20E-09
	GO:0044444	C	cytoplasmic part	7.30E-11	3.50E-09
	GO:0044424	C	intracellular part	1.40E-10	4.50E-09
	GO:0005622	C	intracellular	1.30E-10	4.50E-09
	GO:0043229	C	intracellular organelle	1.40E-09	3.10E-08
	GO:0009536	C	plastid	1.30E-09	3.10E-08
	GO:0043226	C	organelle	1.40E-09	3.10E-08
	GO:0043231	C	intracellular membrane-bounded organelle	1.80E-09	3.20E-08
	GO:0043227	C	membrane-bounded organelle	1.80E-09	3.20E-08
**Cluster 3**	No significant term				
**Cluster 4**	No significant term				
**Cluster 5**	No significant term				
**Cluster 6**	No significant term				
**Cluster 8**	No significant term				
**Cluster 9**	No significant term				
**Cluster 10**	No significant term				
**Cluster 11**	No significant term				

Expression data from Janssen et al. [49] was mined to identify the expression profiles for any genes encoding plastid-targeted protein. Clustering of genes was performed based upon expression profile revealing a set of 11 significant clusters. GO term enrichment was performed to identify significantly enriched terms associated with each cluster. Gene Ontology terms are provided for biological process (P), molecular function (F), and cellular component (C).

**Figure 2:**
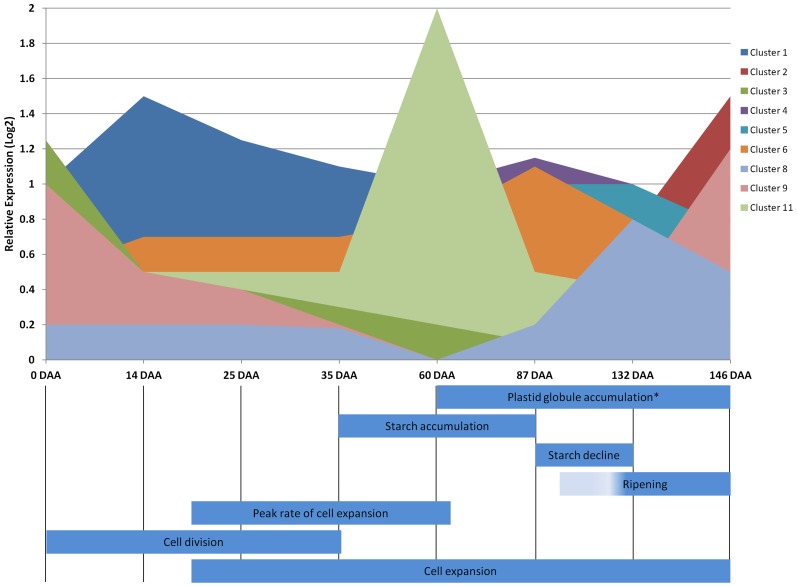
Overlay of apple gene expression clusters with distinct fruit development events. Relative expression of the co-expressed genes encoding plastid-targeted proteins were merged and displayed along significant events occurring within the developmental continuum of apple fruits, adapted from Janssen et al. 2008. An additional event unique to apple fruit plastids, globule accumulation was added as described by Clijsters in 1969.

In order to determine if genes encoding plastid-targeted proteins were indeed expressed within the fruit of apple, data from a previous study were analyzed [49]. The initial microarray experiment was a large scale analysis representing 13,000 of the ∼57,000 apple genes, and was designed around many significant physiological events occurring during apple fruit development. These 13,000 genes were compared to the genes encoding predicted plastid targeted proteins described earlier in this study. About 20% of the genes (2,224 genes) encoding predicted plastid-targeted proteins mapped back to genes represented in the Janssen study. Analysis with MultiExperiment Viewer revealed that the majority of these genes were co-expressed in 9 clusters. To show how these expression profiles may relate to important fruit developmental events, expression profiles for the co-expressed genes were overlaid with those events described in Janssen et al. (Figure 2). An additional event, plastid globule accumulation, was also added, as it was noted in developing apple fruits alongside the unstacking of photosynthetic membranes [54]. As seen in Figure 2, the gene expression of these clusters and their GO terms coincide to some extent with the processes occurring within the apple fruits. Many of the biological process GO terms and KEGG pathways associated with each gene expression cluster suggest that expression of genes encoding plastid-targeted proteins may coincide with these important events. The expression of Cluster 1 greatly mirrors the photosynthetic activity of apple fruit tissue, with highest expression occurring in young, photosynthetically-capable fruit, and expression lowering as the fruit matures and has a reduction in photosynthetic capabilities. Additionally, the expression of those genes in Cluster 2 appear to mirror the development of carotenoids, volatile compounds, and maturation of fruit, with expression lowest in young fruit and increasing as the fruit reaches maturity. In particular the expression of genes whose products are involved in lipid metabolic processes, secondary metabolic processes, biosynthetic processes, and catabolic processes, as determined via GO term enrichment would be great candidates for further study in their participation in apple fruit volatile production. Cluster 11 is particularly interesting as it is comprised of genes whose expression peaks at a single time point (60 DAA), however, the associated KEGG pathways and GO terms do not suggest a connection to the significant fruit processes of cell expansion and starch accumulation occurring at that time point. Blast2GO analysis revealed that 15.2% of the entire plastid-targeted proteome of apple lacked GO term information. However, the set of 2,224 genes represented in this study reveals that this subset is better characterized as it contains only 78 (3.5%) sequences with no associated GO terms. Of course the mere expression of a gene does not indicate that a functional protein is present within the fruit plastids as this process could be affected or controlled at a number of levels including translation, interaction with chaperone proteins, redox state of the plastid, presence of appropriate translocation proteins, protein and mRNA stability and turnover, and likely many other factors. Regardless, the data presented in this study indicate that the expression of genes encoding plastid-targeted proteins is dynamic in the fruit of *Malus × domestica* and may play key roles in the development and quality of apple fruit.

### Prediction and comparative analysis of plastid-targeted proteomes

In order to identify plastid targeted-proteins in seven species of interest (*Arabidopsis thaliana*, *Prunus persica*, *Malus × domestica*, *Populus trichocarpa*, *Fragaria vesca*, *Solanum lycopersicum*, and *Vitis vinifera*), plastid-targeting predictions were primarily performed using Target P. TargetP was selected in order to be consistent with previously published work in this area and because previous studies have found TargetP to be the most reliable single prediction program [17], [20]. TargetP analysis revealed a large variance in the percentage of total transcripts encoding putative plastid-targeted proteins between the investigated species. The largest of these datasets belonged to *Malus × domestica*with 18.3% of its nuclear-encoded proteins predicted to be plastid-targeted, while the lowest was that of *Populus trichocarpa* with only 9.9% (Table 2). Header information for the predicted plastid-targeted datasets is provided in File S3.

**Table 2:** Results of putative plastid-targeted protein prediction.

Species	Reference	Unique predicted protein-coding genes	Predicted to be plastid-targeted
*Arabidopsis thaliana*	[40]	27,416	5,382* (19.6%)
*Malus × domestica* (Apple)	[26]	57,386	10,492 (18.3%)
*Vitis vinifera* (Grapevine)	[22]	26,346	3,015 (11.4%)
*Prunus persica* (Peach)	[21]	28,689	3,860 (13.5%)
*Populus trichocarpa* *(*Black cottonwood)	[25]	45,555	4,515 (9.9%)
*Fragaria vesca *(Woodland strawberry)	[24]	34,809	4,922 (14.1%)
*Solanum lycopersicum* (Tomato)	[23]	33,926	4,009 (11.8%)

Predicted transcripts were selected from 7 sequenced genomes representing both model organisms as well as agriculturally important fruit-producing crops. Translated sequences were analyzed using TargetP to predict cellular localization. Sequences were clustered with 40% coverage and 40% identity to predict proteins unique to the plastid proteome of each species. *Represents sequences from Chloroplast 2010 project and sequences associated with predicted plastid-targeted embryo lethal mutant.

### Comparison of plastid-targeted proteomes with model systems

The predicted plastid proteomes for *Malus × domestica*, *Fragaria vesca*, *Populus trichocarpa*, *Prunus persica*, *Vitis vinifera*, and *Solanum lycopersicum* were independently compared with the *Arabidopsis* plastid proteome dataset as well as the entire *Arabidopsis* protein set using USEARCH. In *Arabidopsis*, the Chloroplast2010 project (www.plastid.msu.edu) [40] identified 5,181 unique genes encoding plastid-targeted proteins using software predictions and direct experimental evidence. Since this dataset did not represent embryo lethal mutants, an additional 201 genes predicted to encode embryo lethal plastid-targeted proteins from the SeedGenes database [55] were added to the dataset used in this study for comparative analysis to bring it to a total of 5,382 sequences. Comparison at 40% identity and 40% coverage (40/40) reveals that about 50% of each predicted plastid proteome has a likely homolog in the *Arabidopsis* plastid-targeted proteome subset, while about 60–70% of the proteins have likely homologs in the entire *Arabidopsis* protein subset (Table 3). Further comparison with 50/50 clustering parameter lowers these estimates significantly.

**Table 3:** Clustering of predicted plastid proteomes with *Arabidopsis* datasets.

Species	Predicted plastid- targeted proteins	Percent clustered with *At* chloroplast (40/40)	Percent clustered with all *At* proteins (40/40)	Percent clustered with *At* chloroplast (50/50)	Percent clustered with all *At* proteins (50/50)
***Arabidopsis thaliana***	5,382*	100%	100%	100%	100%
***Malus × domestica***	10,492	40.7%	56.7%	32.2%	44.5%
***Vitis vinifera***	3,015	57.3%	77.0%	49.5%	65.8%
***Prunus persica***	3,860	59.9%	76.9%	52.0%	67.3%
***Populus tichocarpa***	4,515	51.8%	68.3%	44.5%	58.4%
***Fragaria vesca***	4,922	40.9%	56.1%	33.3%	45.0%
***Solanum lycopersicum***	4,009	55.2%	69.7%	45.7%	57.0%

Predicted plastid-targeted proteins unique to each species were clustered against the peptide sequences derived from the Chloroplast 2010 dataset with the addition of embryo lethal sequences (*At* chloroplast) as well as the entire *Arabidopsis* protein set from TAIR V10 (all *At* proteins) using USEARCH. Clustering was performed using the parameters of 40% coverage with 40% identity (40/40) as well as 50% coverage with 50% identity (50/50).

Additional comparison of putative plastid-targeted protein sequences for all six species was performed against the plastid-targeted proteome of *Solanum lycopersicum*, another model system for plastid biology research. This analysis showed that smaller proportions of the plastid proteomes had homologs in the tomato plastid proteome (ranging from 32–48%) than they had in the Arabidopsis proteome (40–60%) (Table 4). Strawberry and apple had the lowest similarity with the predicted plastid-proteomes of both *Arabidopsis* and tomato, while peach had the highest.

**Table 4:** USEARCH-based matching of unique plastid-targeted proteins.

Species	Predicted plastid- targeted Proteins	Number Clustered with *At* Chloroplast (40/40)	Number Clustered with *Sl* Chloroplast (40/40)	Proteins clustered with Cp Proteins from 6 Other Species	Unique Plastid- targeted Proteins
*Arabidopsis thaliana*	5,382*	5,382 (100%)	2,503 (46.5%)	4,099 (76.2%)	1,446 (26.9%)
*Malus × domestica*	10,492	4,265 (40.7%)	3,442 (32.8%)	6,235 (59.4%)	4,257 (40.6%)
*Vitis vinifera*	3,015	1,729 (57.3%)	1,396 (46.3%)	2,265 (75.1%)	750 (24.9%)
*Prunus persica*	3,860	2,312 (59.9%)	1,857 (48.1%)	3,232 (83.7%)	628 (16.3%)
*Populus tichocarpa*	4,515	2,340 (51.8%)	1,949 (43.2%)	3,207 (71.0%)	1,308 (29.0%)
*Fragaria vesca*	4,922	2,014 (40.9%)	1,584 (32.2%)	2,880 (58.5%)	2,042 (41.5%)
*Solanum lycopersicum*	4,009	2,213 (55.2%)	4,009 (100%)	2,799 (69.8%)	1,210 (30.2%)

Datasets comprised of putative plastid-targeted proteins unique to each species were clustered against the predicted plastid-targeted protein sequences of *Arabidopsis thaliana* (*At)* from Chloroplast2010 and TAIR V10, *Solanum lycopersicum* (*Sl)*, and a database consisting of the plastid-targeted proteins from all 6 species using USEARCH. Sequences were clustered globally with 40% coverage with 40% identity (40/40). Results suggest that a large portion of the plastid-targeted proteins may be unique to each respective species.

### Identification of unique plastid-targeted proteins

**Figure 3:**
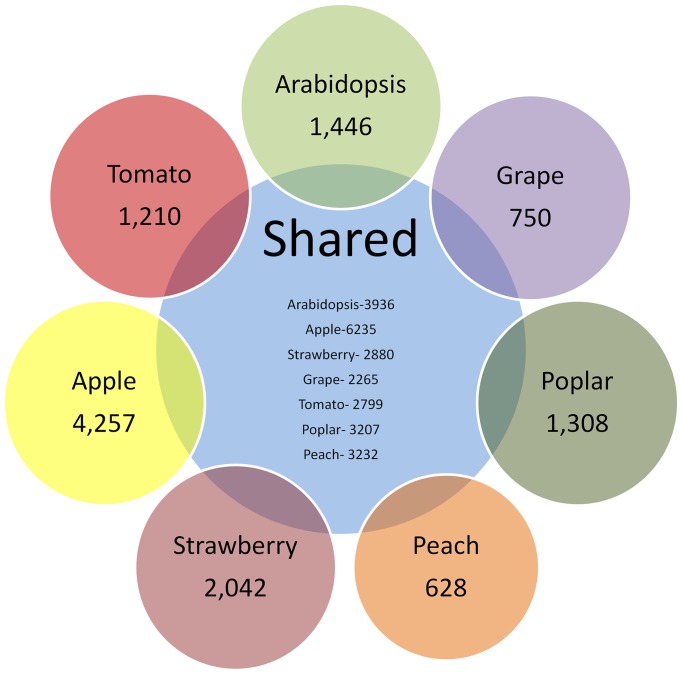
Comparison of predicted plastid-targeted proteomes across seven sequenced genomes. Protein sequences generated from the genomes of *Arabidopsis thaliana*, *Vitis vinifera*, *Populus trichocarpa*, *Prunus persica*, *Fragaria vesca*, *Malus × domestica*, and *Solanum lycopersicum* were clustered at 40 percent identity and 40 percent coverage using USEARCH. Comparison reveals large subsets for each species appearing to be unique to each species' respective plastids.

Two separate analyses were performed to identify the plastid-targeted proteins in each of the seven species examined. The first consisted of a USEARCH-based comparison of predicted plastid-targeted proteins against the plastid-targeted protein sequences from the other six species. A second comparison utilized a clustering technique with UCLUST [33]. In this analysis the plastid-targeted proteins from all species were clustered together and clusters containing singletons or sequences from a single species were identified and further analyzed. In both the USEARCH and UCLUST-based analyses, a significant proportion of each predicted plastid proteome was found to be unique to that species (Table 5). The proportion of uniquely targeted proteins ranges from 16.3% in *Prunus persica* to 41.5% in *Fragaria vesca* in the USEARCH method and 20.6% in *Prunus persica* to 46.8% in *Fragaria vesca* in the UCLUST method. However, the majority of the protein sequences have a homolog in at least one of the investigated species (Figure 3). In order to determine if uniquely-targeted proteins were in fact completely unique to each species, as opposed to alternatively targeted, a comparison with USEARCH 40/40 parameters was performed against datasets comprising the entire predicted proteomes of the other 6 species (Table 5). This investigation revealed thata significant number of proteins that lacked homology with interspecies plastid-targeted proteins, in fact have alternatively targeted homologs. This difference was most significant in *Arabidopsis* and *Malus × domestica* increasing the percentage of homology by 15.3% and 16.8%, respectively. Roughly 7–10% more proteins had homologs when using this matching scheme in the other investigated species. Information on these uniquely plastid-targeted proteins is provided in File S4.

**Table 5:** Uniquely targeted plastid-targeted protein sequences.

Species	Predicted plastid-targeted Proteins	Unique to Species (USEARCH 40/40)	Unique to Species (UCLUST50)	Number Clustered with All Proteins from 6 Other Species (USEARCH 40/40)
***Arabidopsis thaliana***	5,382*	1,446 (26.9%)	2,154 (40.0%)	4,928 (91.6%)
***Malus × domestica***	10,492	4,257 (40.6%)	4,787 (45.6%)	7,911 (76.2%)
***Vitis vinifera***	3,015	750 (24.8%)	976 (32.3%)	2,561 (84.9%)
***Prunus persica***	3,860	628 (16.3%)	795 (20.6%)	3,506 (90.8%)
***Populus tichocarpa***	4,515	1,308 (29.0%)	1,732 (38.4%)	3,630 (80.4%)
***Fragaria vesca***	4,922	2,042 (41.5%)	2,305 (46.8%)	3,338 (67.8%)
***Solanum lycopersicum***	4,009	1,210 (30.2%)	1,654 (41.3%)	3,122 (77.9%)

A USEARCH comparison of plastid-targeted protein datasets was performed at 40% identity and 40% coverage against a database containing the chloroplast protein sequences from all six other species investigated in this study. A second comparison was performed against a database containing the entire protein set from the other species. An increase in matching suggests the presence of differentially localized homologues in other systems. Additionally, these results suggest a sizeable number of plastid-targeted proteins may be unique to each species.

GO term enrichment analysis was performed on the species-specific plastid-targeted protein sequences for both UCLUST and USEARCH-based analyses. Comparisons were performed using a Fisher's test with the entire predicted plastid proteome as a reference. This analysis revealed significantly enriched GO terms for both comparative techniques. In the USEARCH based technique, the majority of enriched GO terms were present in the unique apple plastid-targeted sequences with the most significant GO terms were DNA metabolic process (GO:006259, p-value 8.30E-60) and cellular macromolecule metabolic process (GO:0044260) (Table 6). Additionally, the GO term nucleic acid binding (GO:0003676) was enriched in poplar as well as apple. No significant GO terms were found for grape, strawberry or tomato. More GO terms were found to be enriched in the UCLUST-based comparison (Table 7). In this analysis transcription regulator activity (GO:0030528) and transcription factor activity (GO:0003700) were enriched in the *Arabidopsis* and tomato datasets. DNA binding (GO:0003677) was enriched in apple, *Arabidopsis* and tomato. Cell death (GO:0008219) and death (GO:0016265) were also enriched in the apple UCLUST50 dataset.

**Table 6:** GO terms enriched in uniquely targeted proteins as determined by USEARCH 40/40 method.

	GO term	Ontology	Description	p-value	FDR
**Apple**	GO:0006259	P	DNA metabolic process	8.30E-60	3.30E-57
	GO:0044260	P	cellular macromolecule metabolic process	4.20E-14	8.40E-12
	GO:0006807	P	nitrogen compound metabolic process	1.60E-12	1.60E-10
	GO:0006139	P	nucleobase, nucleoside, nucleotide and nucleic acid metabolic process	1.60E-12	1.60E-10
	GO:0043170	P	macromolecule metabolic process	2.90E-11	2.30E-09
	GO:0003676	F	nucleic acid binding	1.30E-53	1.60E-51
	GO:0005488	F	Binding	2.80E-24	1.70E-22
	GO:0003677	F	DNA binding	8.30E-05	3.30E-03
**Arabidopsis**	GO:0005634	C	Nucleus	8.30E-16	1.70E-13
**Grapevine**	No significant term				
**Peach**	GO:0016787	F	hydrolase activity	1.60E-06	1.10E-04
**Black cottonwood**	GO:0003676	F	nucleic acid binding	2.90E-06	3.40E-04
**Strawberry**	No significant term				
**Tomato**	No significant term				

GO terms were determined for all predicted plastid-targeted proteomes. Enrichment of GO terms was determined with agriGO for the subset of plastid-targeted proteins unique to each species compared to the entire plastid proteome. Gene Ontology terms are provided for biological process (P), molecular function (F), and cellular component (C).

**Table 7:** GO terms enriched in uniquely plastid-targeted proteins identified with UCLUST 50% method.

	GO term	Ontology	Description	p-value	FDR
**Apple**	GO:0006259	P	DNA metabolic process	4.60E-08	1.80E-05
	GO:0016265	P	death	2.90E-04	3.80E-02
	GO:0008219	P	cell death	2.90E-04	3.80E-02
	GO:0003676	F	nucleic acid binding	3.90E-15	4.90E-13
	GO:0003677	F	DNA binding	2.30E-12	1.40E-10
	GO:0005488	F	binding	2.20E-10	9.30E-09
**Arabidopsis**	GO:0030528	F	transcription regulator activity	7.20E-06	3.60E-04
	GO:0003700	F	transcription factor activity	6.90E-06	3.60E-04
	GO:0003677	F	DNA binding	9.10E-05	3.00E-03
	GO:0005634	C	nucleus	1.30E-25	2.70E-23
	GO:0005654	C	nucleoplasm	6.60E-06	6.70E-04
	GO:0031981	C	nuclear lumen	3.90E-05	1.30E-03
	GO:0031974	C	membrane-enclosed lumen	3.90E-05	1.30E-03
	GO:0043233	C	organelle lumen	3.90E-05	1.30E-03
	GO:0070013	C	intracellular organelle lumen	3.90E-05	1.30E-03
	GO:0044428	C	nuclear part	1.10E-04	2.60E-03
	GO:0044446	C	intracellular organelle part	1.10E-04	2.60E-03
	GO:0044422	C	organelle part	1.10E-04	2.60E-03
**Grapevine**	No significant term				
**Peach**	GO:0016787	F	hydrolase activity	1.20E-04	8.90E-03
**Black cottonwood**	GO:0003676	F	nucleic acid binding	3.60E-04	4.40E-02
**Strawberry**	No significant term				
**Tomato**	GO:0003677	F	DNA binding	8.60E-05	6.60E-03
	GO:0030528	F	transcription regulator activity	2.60E-04	1.00E-02
	GO:0003700	F	transcription factor activity	4.70E-04	1.20E-02

Blast2GO was used to determine GO terms associated with all predicted plastid-targeted proteins. Enrichment analysis was performed with agriGO to identify significant enriched GO terms. Gene Ontology terms are provided for biological process (P), molecular function (F), and cellular component (C).

While a significant amount of functional annotation was performed with Blast2GO, a large proportion of the proteins predicted to be unique to each species lack any associated GO term (Table 8). Proteins predicted to be unique to the plastids of *Arabidopsis* appear to be the best characterized with 98% containing some form of GO information, followed by those of *Prunus persica* with 81.9%. *Solanum lycopersicum* displays the least amount of GO information with only 56.5% of these uniquely plastid-targeted proteins having associated GO terms.

**Table 8:** Percentage of unique plastid proteome containing GO information.

Species	Plastid-targeted Proteins	Number w/o GO information	Unique to Species (USEARCH)	Number w/o GO information	Unique to Species (UCLUST)	Number w/o GO information
***Arabidopsis thaliana***	5,382	10 (0.2%)	1,446	6 (0.4%)	2,154	8 (0.4%)
***Malus × domestica***	10,492	1,592 (15.2%)	4,257	1,348 (31.7%)	4,817	1,386 (28.8%)
***Vitis vinifera***	3,015	542 (18.0%)	750	415 (55.3%)	976	455 (46.6%)
***Prunus persica***	3,860	719 (18.6%)	628	379 (60.4%)	795	450 (56.6%)
***Populus tichocarpa***	4,515	527 (11.7%)	1,308	425 (32.5%)	1,732	462 (26.6%)
***Fragaria vesca***	4,922	1,539 (31.3%)	2,042	1,350 (66.1%)	2,305	1,385 (60.1%)
***Solanum*** ***lycopersicum***	4,009	873 (21.8%)	1,210	693 (57.3%)	1,654	750 (45.3%)

Plastid-targeted proteins were analyzed using Blast2GO to identify GO terms associated with each protein sequence. With the exception of *Arabidopsis,* significant proportions of chloroplast-targeted proteins datasets lack GO term information. This further increases in the datasets comprised of chloroplast-targeted proteins unique to each investigated species.

### Analysis of proteins conserved between all plastid-targeted proteomes

Two separate analyses were performed to identify proteins which were predicted to be targeted to the plastids of all seven angiosperms studied. First analysis workflow utilized a semi-global approach where UCLUST [33] was used to cluster the proteins of all predicted plastid proteomes at 50% identity. The second approach utilized a global approach where the plastid proteome of *Arabidopsis thaliana* was compared with every other species’ predicted plastid proteome at 40% identity and 40% coverage. Those proteins which have a matched protein from all other six species were then determined to be conserved across the plastid proteomes.

**Figure 4:**
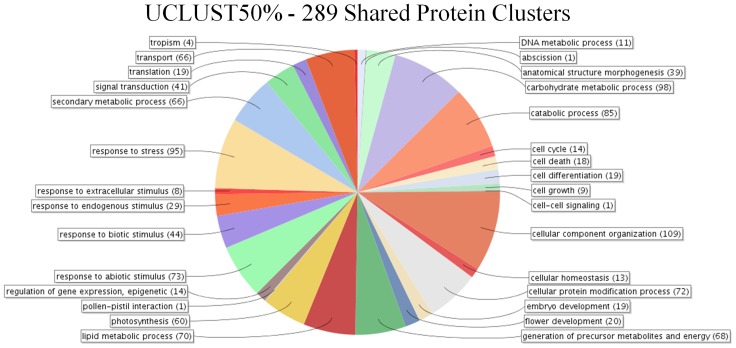
Biological process GO term composition of proteins shared between the 7 investigated plastid-targeted proteomes using two separate techniques. Two separate analyses were performed to identify proteins within the predicted plastid-targeted proteomes of seven species. The first, UCLUST with 50% identity, generated 15,750 clusters, 289 of which contained a member from all 7 species. USEARCH comparison performed at 40% identity and 40% coverage identified 737 sequences in the Arabidopsis thaliana putative plastid-targeted dataset which had a match in a protein sequence from the plastid-targeted sequences from all of the other 6 species. Sequences were analyzed via Blast2GO to determine the biological processes in which they partake.

**Figure 5:**
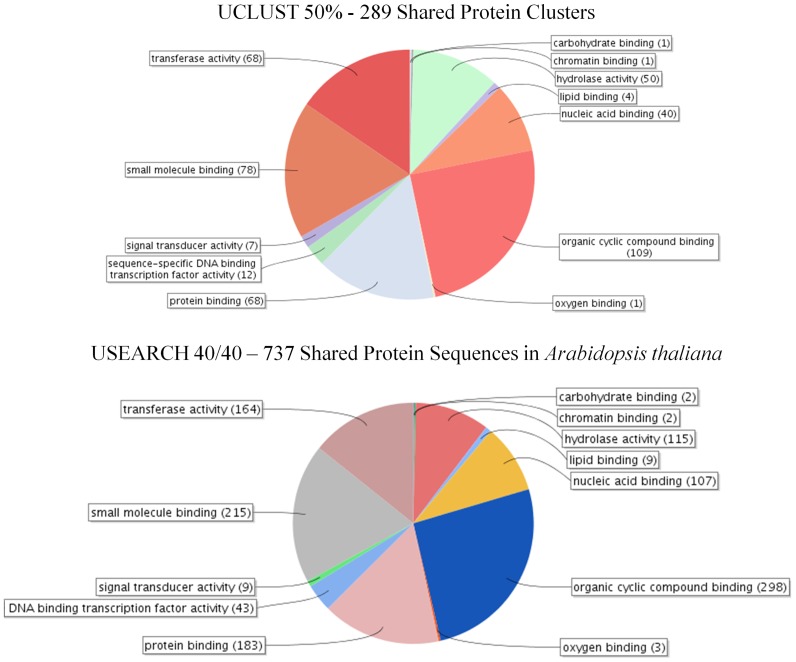
Molecular Function GO term composition of proteins shared between the 7 investigated plastid-targeted proteomes using two separate techniques. Two separate analyses were performed to identify proteins within the predicted plastid-targeted proteomes of seven species. The first, UCLUST with 50% identity, generated 15,750 clusters, 289 of which contained a member from all 7 species. USEARCH comparison performed at 40% identity and 40% coverage identified 737 sequences in the Arabidopsis thaliana putative plastid-targeted dataset which had a match in a protein sequence from the plastid-targeted sequences from all of the other 6 species. Sequences were analyzed via Blast2GO to determine the molecular functions of proteins at level 3.

The first analysis using UCLUST at 50% identity identified 289 clusters of proteins which had at least one member from all seven species. These 289 clusters contain 497 unique sequences from *Arabidopsis thaliana*, 773 from *Malus × domestica*, 384 from *Vitis vinifera*, 392 from *Fragaria vesca*, 545 from *Populus trichocarpa*, 439 from *Prunus persica*, and 478 from *Solanum lycopersicum*. Blast2GO analysis reveals that these proteins are involved in a large number of biological processes (Figure 4) with the most populous being cellular component organization (GO:0016043, 109 proteins), carbohydrate metabolic process (GO:0005975, 98 proteins), and response to stress (GO:0006950, 95 proteins). The electron transport chain-related molecular functions for this data set reveals that the majority of proteins fit into six main categories; organic cyclic compound binding (GO:0097159 109 proteins), small molecule binding (GO:0036094 78 proteins), transferase activity (GO:0016740 68 proteins), protein binding (GO:0005515 68 proteins), hydrolase activity (GO:0016787 50 proteins), and nucleic acid binding (GO:0003676 40 proteins) (Figure 5). GO term enrichment was performed by selecting a single *Arabidopsis thaliana* protein sequence and utilizing agriGO to compare with those GO terms from the entire *Arabidopsis thaliana* predicted plastid-targeted proteome. This dataset contains 56 GO terms which were enriched with a p-value cut-off of 0.01 (Table 9). The lowest p-values are associated with the GO terms photosynthesis (GO:0015979), carbohydrate metabolic process (GO:0005975), macromolecule modification (GO:0043412), protein modification process (GO:0006464) and thylakoid (GO:0009579) respectively.

**Table 9:** GO terms enriched in *Arabidopsis thaliana* members of the 289 plastid-targeted protein clusters shared between all species investigated.

GO term	Ontology	Description	Number in UCLUST50	Number in At Cp-targeted Proteome	p-value	FDR
GO:0015979	P	photosynthesis	96	362	1.30E-22	5.10E-20
GO:0005975	P	carbohydrate metabolic process	167	911	3.70E-19	7.40E-17
GO:0043412	P	macromolecule modification	138	718	2.10E-17	2.10E-15
GO:0006464	P	protein modification process	138	718	2.10E-17	2.10E-15
GO:0008152	P	metabolic process	419	3515	1.90E-16	1.50E-14
GO:0006091	P	generation of precursor metabolites and energy	108	515	2.40E-16	1.60E-14
GO:0044238	P	primary metabolic process	388	3152	4.60E-16	2.60E-14
GO:0009987	P	cellular process	436	3796	9.90E-15	4.90E-13
GO:0044267	P	cellular protein metabolic process	157	925	1.40E-14	6.70E-13
GO:0016265	P	death	54	203	7.10E-13	2.60E-11
GO:0008219	P	cell death	54	203	7.10E-13	2.60E-11
GO:0019748	P	secondary metabolic process	112	624	8.60E-12	2.80E-10
GO:0009628	P	response to abiotic stimulus	148	940	6.80E-11	2.10E-09
GO:0019538	P	protein metabolic process	178	1238	8.30E-10	2.40E-08
GO:0006629	P	lipid metabolic process	116	703	1.00E-09	2.70E-08
GO:0016043	P	cellular component organization	181	1274	1.50E-09	3.30E-08
GO:0006519	P	cellular amino acid and derivative metabolic process	130	824	1.50E-09	3.30E-08
GO:0044281	P	small molecule metabolic process	130	824	1.50E-09	3.30E-08
GO:0050896	P	response to stimulus	220	1657	3.90E-09	8.10E-08
GO:0009058	P	biosynthetic process	293	2394	4.40E-09	3.80E-08
GO:0044237	P	cellular metabolic process	316	2637	4.20E-09	3.80E-08
GO:0006950	P	response to stress	177	1274	1.60E-08	2.80E-07
GO:0023052	P	signaling	99	636	5.40E-07	9.40E-06
GO:0007165	P	signal transduction	86	534	8.80E-07	1.30E-05
GO:0050794	P	regulation of cellular process	86	534	8.80E-07	1.30E-05
GO:0009056	P	catabolic process	146	1056	8.80E-07	1.30E-05
GO:0065007	P	biological regulation	135	959	1.00E-06	1.50E-05
GO:0023046	P	signaling process	87	545	1.10E-06	1.50E-05
GO:0023060	P	signal transmission	87	545	1.10E-06	1.50E-05
GO:0050789	P	regulation of celbiological process	102	674	1.40E-06	1.80E-05
GO:0044260	P	cellular macromolecule metabolic process	194	1530	2.60E-06	3.30E-05
GO:0006810	P	transport	136	1010	1.10E-05	1.30E-04
GO:0051234	P	establishment of localization	136	1010	1.10E-05	1.30E-04
GO:0051179	P	localization	136	1010	1.10E-05	1.30E-04
GO:0009605	P	reponse to external stimulus	57	342	3.50E-05	4.00E-04
GO:0006807	P	nitrogen compound metabolic process	203	1686	4.70E-05	5.00E-04
GO:0006139	P	nucleobase, nucleoside, nucleotide and nucleic acid metabolic process	203	1686	4.70E-05	5.00E-04
GO:0009607	P	response to biotic stimulus	88	618	1.10E-04	1.10E-03
GO:0043170	P	macromolecule metabolic process	216	1842	1.20E-04	1.20E-03
GO:0009719	P	reponse to endogenous stimulus	69	484	7.40E-04	7.40E-03
GO:0003824	F	catalytic activity	291	2207	4.00E-13	4.40E-11
GO:0000166	F	nucleotide binding	139	934	1.80E-08	1.00E-06
GO:0016740	F	transferase activity	115	760	2.20E-07	4.80E-06
GO:0016301	F	kinase activity	56	286	1.80E-07	4.80E-06
GO:0016772	F	transferase activity, transferring phosphorus-containing groups	56	286	1.80E-07	4.80E-06
GO:0005488	F	binding	327	2878	9.30E-07	1.70E-05
GO:0016817	F	hydrolase activity, acting on acid anhydrides	11	31	1.30E-04	1.30E-03
GO:0016818	F	hydrolase activity, acting on acid anhydrides, in phosphorus-containing anhydrides	11	31	1.30E-04	1.30E-03
GO:0003774	F	motor activity	11	31	1.30E-04	1.30E-03
GO:0016462	F	pyrophosphatase activity	11	31	1.30E-04	1.30E-03
GO:0017111	F	nucleoside-triphosphatase activity	11	31	1.30E-04	1.30E-03
GO:0009579	C	thylakoid	111	524	3.50E-17	6.90E-15
GO:0009536	C	plastid	337	2749	1.10E-11	1.10E-09
GO:0016020	C	membrane	228	1712	1.10E-09	7.10E-08
GO:0044444	C	cytoplasmic part	394	3569	4.70E-08	2.30E-06
GO:0005737	C	cytoplasm	409	3823	1.10E-06	4.40E-05

GO terms from the 497 *Arabidopsis thaliana* proteins present within the 289 shared clusters were analyzed by agriGO to identify enriched GO terms. Chi-square test was performed with a p-value cutoff of 0.01.

Analysis with USEARCH 40/40 identified the presence of 737 unique protein sequences from Arabidopsis thaliana with a matching protein in the predicted plastid-targeted proteomes of *Solanum lycopersicum*, *Prunus persica*, *Vitis vinifera*, *Malus × domestica*, *Fragaria vesca*, and *Populus trichocarpa*. As in the UCLUST50 analysis, the top three biological processes in this dataset as determined by Blast2GO are cellular component organization (GO:0016043, 254 proteins), response to stress (GO:0006950, 251 proteins), and carbohydrate metabolic process (GO:0005975, 210 proteins) (Figure 4). Again, the most populous molecular function GO terms mirror those in the UCLUST 50% with the majority falling into the six categories of: organic cyclic compound binding (GO:0097159 298 proteins), small molecule binding (GO:0036094 215 proteins), transferase activity (GO:0016740 164 proteins), protein binding (GO:0005515 183 proteins), hydrolase activity (GO:0016787 115 proteins), and nucleic acid binding (GO:0003676 107 proteins) (Figure 5). GO term enrichment using agriGO identified 59 GO terms to be enriched with a p-value cut-off of 0.01 (Table 10). The lowest p-values are associated with the GO terms photosynthesis (GO:0015979), thylakoid (GO:0009579), macromolecule modification (GO:0043412), protein modification process (GO:0006464), and generation of precursor metabolites and energy respectively (GO:0006091).

**Table 10:** GO terms enriched in *Arabidopsis thaliana* members of the 737 proteins with a match in all 6 species as determined by USEARCH4040.

GO Term	Ontology	Description	Number in USEARCH 4040	Number in At Cp-targeted Proteome	p-value	FDR
GO:0015979	P	photosynthesis	127	362	8.90E-22	3.50E-19
GO:0043412	P	macromolecule modification	190	718	7.70E-18	1.00E-15
GO:0006464	P	protein modification process	190	718	7.70E-18	1.00E-15
GO:0006091	P	generation of precursor metabolites and energy	146	515	4.70E-16	3.70E-14
GO:0009987	P	cellular process	634	3796	4.50E-16	3.70E-14
GO:0008152	P	metabolic process	598	3513	1.80E-15	1.20E-13
GO:0044238	P	primary metabolic process	551	3152	4.30E-15	2.40E-13
GO:0044267	P	cellular protein metabolic process	218	925	7.60E-15	3.80E-13
GO:0005975	P	carbohydrate metabolic process	210	911	3.00E-13	1.30E-11
GO:0044237	P	cellular metabolic process	471	2637	1.50E-12	5.90E-11
GO:0009628	P	response to abiotic stimulus	212	940	1.90E-12	6.90E-11
GO:0006519	P	cellular amino acid and derivative metabolic process	191	824	3.60E-12	1.10E-10
GO:0044281	P	small molecule metabolic process	191	824	3.60E-12	1.10E-10
GO:0050896	P	response to stimulus	316	1657	6.00E-10	1.70E-08
GO:0019538	P	protein metabolic process	249	1238	1.40E-09	3.70E-08
GO:0016043	P	cellular component organization	254	1274	2.00E-09	4.60E-08
GO:0065007	P	biological regulation	203	959	2.00E-09	4.60E-08
GO:0006950	P	response to stress	251	1274	8.20E-09	1.80E-07
GO:0044260	P	cellular macromolecule metabolic process	288	1530	2.70E-08	5.70E-07
GO:0016265	P	death	61	203	6.30E-08	1.20E-06
GO:0008219	P	cell death	61	203	6.30E-08	1.20E-06
GO:0019748	P	secondary metabolic process	137	624	3.00E-07	5.40E-06
GO:0009058	P	biosynthetic process	407	2394	5.50E-07	9.50E-06
GO:0048856	P	anatomical structure development	155	749	1.40E-06	2.40E-05
GO:0065008	P	regulation of biological quality	84	344	2.00E-06	3.10E-05
GO:0006629	P	lipid metabolic process	146	703	2.70E-06	4.10E-05
GO:0043170	P	macromolecule metabolic process	318	1842	1.40E-05	2.00E-04
GO:0050789	P	regulation of biological process	134	674	6.70E-05	9.50E-04
GO:0009607	P	response to biotic stimulus	124	618	9.40E-05	1.30E-03
GO:0009653	P	anatomical structure morphogenesis	117	584	1.70E-04	2.30E-03
GO:0022414	P	reproductive process	86	404	2.50E-04	3.10E-03
GO:0007165	P	signal transduction	107	534	3.60E-04	4.30E-03
GO:0050794	P	regulation of cellular process	107	534	3.60E-04	4.30E-03
GO:0023046	P	signaling process	108	545	5.00E-04	5.60E-03
GO:0023060	P	signal transmission	108	545	5.00E-04	5.60E-03
GO:0006810	P	transport	181	1010	6.20E-04	6.50E-03
GO:0051234	P	establishment of localization	181	1010	6.20E-04	6.50E-03
GO:0051179	P	localization	181	1010	6.20E-04	6.50E-03
GO:0090066	P	regulation of anatomical structure size	46	191	8.60E-04	8.20E-03
GO:0016049	P	cell growth	46	191	8.60E-04	8.20E-03
GO:0008361	P	regulation of cell size	46	191	8.60E-04	8.20E-03
GO:0032535	P	regulation of cellular component size	46	191	8.60E-04	8.20E-03
GO:0023052	P	signaling	121	636	9.70E-04	9.00E-03
GO:0042592	P	homeostatic process	40	161	1.10E-03	9.90E-03
GO:0019725	P	cellular homeostasis	40	161	1.10E-03	9.90E-03
GO:0016301	F	kinase activity	95	286	1.10E-14	6.10E-13
GO:0016772	F	transferase activity, transferring phosphorus-containing groups	95	286	1.10E-14	6.10E-13
GO:0000166	F	nucleotide binding	215	934	1.50E-13	5.60E-12
GO:0003824	F	catalytic activity	406	2207	1.10E-11	3.10E-10
GO:0005488	F	binding	497	2878	4.20E-11	9.30E-10
GO:0016740	F	transferase activity	164	760	3.90E-08	7.20E-07
GO:0016818	F	hydrolase activity, acting on acid anhydrides, in phosphorus-containing anhydrides	17	31	2.70E-06	2.70E-05
GO:0016817	F	hydrolase activity, acting on acid anhydrides	17	31	2.70E-06	2.70E-05
GO:0003774	F	motor activity	17	31	2.70E-06	2.70E-05
GO:0016462	F	pyrophosphatase activity	17	31	2.70E-06	2.70E-05
GO:0017111	F	nucleoside-triphosphatase activity	17	31	2.70E-06	2.70E-05
GO:0009579	C	thylakoid	154	524	1.90E-18	3.70E-16
GO:0016020	C	membrane	321	1712	2.70E-09	2.60E-07
GO:0009536	C	plastid	460	2749	1.50E-07	1.00E-05

GO terms from one *Arabidopsis thaliana* proteins from each of the 737 shared protein sequences were analyzed by agriGO to identify enriched GO terms. Chi-square test was performed with a p-value cutoff of 0.01.

Comparing the USEARCH40/40 dataset along with that of the UCLUST50 dataset reveals that the two methods agree upon 439 shared plastid-targeted sequences, with 58 present only in UCLUST50 and 298 in USEARCH40/40. Both datasets have similar enriched GO terms with 49 enriched compared to the entire *Arabidopsis thaliana* plastid-targeted proteomes. However, USEARCH40/40 enriched GO terms contain an additional 10 biological process not represented in the UCLUST50 analysis; anatomical structure development (GO:0009653), regulation of biological quality (GO:0065008), anatomical structure morphogenesis (GO:0009653), reproductive process (GO:0022414), cell growth (GO:0016049), regulation of anatomical structure size (GO:0090066), regulation of cell size (GO:0008361), regulation of cellular component size (GO:0032535), homeostatic process (GO:0042592), and cellular homeostasis (GO:0019725).

The UCLUST50 enriched GO terms include five biological processes [catabolic process (GO:0009056), response to external stimulus (GO:0009719), nitrogen compound metabolic process (GO:0006807), nucleobase, nucleoside, nucleotide and nucleic acid metabolic process (GO:0006139), and response to endogenous stimulus (GO:0009719)] and two cellular components [cytoplasmic part (GO:0044444) and cytoplasm (GO:0005737)] not represented in the USEARCH40/40 analysis. A complete list of the 478 *Arabidopsis thaliana* loci from the UCLUST50 dataset and the 737 protein sequences from the USEARCH40/40 dataset along with their respective GO terms are provided in File S5.

Proteins conserved in this study were further compared with those from GreenCut2. GreenCut2 represents a collection of 597 nuclear-encoded proteins determined to be conserved across 20 photosynthetic eukaryotes, but absent in non-photosynthetic organisms [56]. A total of 677 unique loci from *Arabidopsis thaliana* (33 redundant of the original set of 710) were compared with those identified as shared between the seven species examined in this study both with USEARCH 40/40, as well as UCLUST 50% using Venny [57]. This comparison identified 70 proteins present in all three datasets, and a substantial set unique to each dataset. Sequence information from this comparison is provided in File S6.

In order to look at the overlap of homologous proteins between each species, the Jaccard similarity coefficient was determined for the USEARCH40/40 (Table 11). This comparison displays that the plastid-targeted proteomes of apple, strawberry, Arabidopsis and poplar are most similar to that of Peach, while the predicted plastid-targeted proteomes of peach, tomato and grape are most similar to *Arabidopsis.* An additional modified Jaccard similarity coefficient matrix was generated with the UCLUST50 analysis. In this matrix the predicted plastid-targeted of all species are most similar to that of peach, while that of peach is most similar to apple.

**Table 11:** Jaccard’s similarity coefficient matrix for seven species based on predicted plastid-targeted proteomes.

USEARCH 40/40							
	Strawberry	Arabidopsis	Apple	Peach	Tomato	Poplar	Grape
**Strawberry**	1.000	0.409	0.365	0.449	0.369	0.322	0.335
**Arabidopsis**	0.409	1.000	0.391	0.466	0.447	0.378	0.397
**Apple**	0.365	0.391	1.000	0.423	0.342	0.310	0.303
**Peach**	0.449	0.466	0.423	1.000	0.427	0.389	0.393
**Tomato**	0.369	0.447	0.342	0.427	1.000	0.352	0.365
**Poplar**	0.322	0.378	0.310	0.389	0.352	1.000	0.330
**Grape**	0.335	0.397	0.303	0.393	0.365	0.330	1.000

UCLUST50							
	Strawberry	Arabidopsis	Apple	Peach	Tomato	Poplar	Grape
**Strawberry**	1.000	0.194	0.199	0.267	0.171	0.158	0.173
**Arabidopsis**	0.194	1.000	0.192	0.253	0.222	0.193	0.210
**Apple**	0.199	0.192	1.000	0.268	0.162	0.156	0.164
**Peach**	0.267	0.253	0.268	1.000	0.229	0.224	0.240
**Tomato**	0.171	0.222	0.162	0.229	1.000	0.176	0.198
**Poplar**	0.158	0.193	0.156	0.224	0.176	1.000	0.184
**Grape**	0.173	0.210	0.164	0.240	0.198	0.184	1.000

Similarity between each species’ plastid-targeted proteomes was represented by calculating the Jaccard coefficient from the UCLUST50 comparison and a USEARCH 4040 analysis.

## Discussion

This study reveals several interesting aspects about the constitution of predicted plastid-targeted proteomes for the species analyzed. A large portion of a plant’s nuclear genome is dedicated to plastid-targeted proteins, many of which lack identity to plastid-targeted proteins in other species. Some plastid-targeted proteins have identity with potentially alternatively-targeted proteins in other systems. Of the predicted plastid-targeted proteins in *Arabidopsis*, 737 have significant identity to predicted plastid-targeted proteins in each of six investigated species, suggesting an evolutionarily core conserved set of plastid-targeted proteins. The caveat is that TargetP accuracy is not well defined, and has been shown to differ between experiments [58]. van Wijk and Baginsky determined that TargetP has a 35% false positive rate, suggesting that the predicted datasets established here will be greatly reduced upon future confirmatory experiments. Experimental approaches to characterize the proteome of plastids or some of their constituents have relied largely upon mass spectrometry techniques [59], [60], [61], [62]. A study in 2004 focused on the isolation and classification of the constituents of the *Arabidopsis thaliana* chloroplast proteome resulting in the identification of 604 nuclear-encoded proteins [60]. However, TargetP (at that time) was only able to correctly predict the plastid localization of 62.3% of these proteins, with 6.1% predicted to target the mitochondria, 8.1% secreted, and 23.5% predicted to have “any other location”. When excluding the envelope proteins, TargetP chloroplast localization accuracy increased to 67.2%. An additional study which identified 241 stromal proteins from *Arabidopsis thaliana* chloroplasts identified through MALDITOF MS and nano-LC-ESI-MS/MS had a much higher predictability to be chloroplast targeted by TargetP with 88% accuracy [61]. Yet another study of 916 nuclear-encoded *Arabidopsis* plastid-targeted proteins revealed that 86% were correctly predicted using TargetP [63]. Such studies indicate that localization prediction methods need to be improved. Some reasons for this could include the complexities of the experimental system, sequence data, dual targeting, splice variants, presence of lesser characterized transport systems, or simply a lack of understanding of mechanisms of localization. As genomes become better characterized and targeting prediction improves, our ability to better understand the commonalities and diversities of plastid compositions and functions will also likely improve.

**Figure 6:**
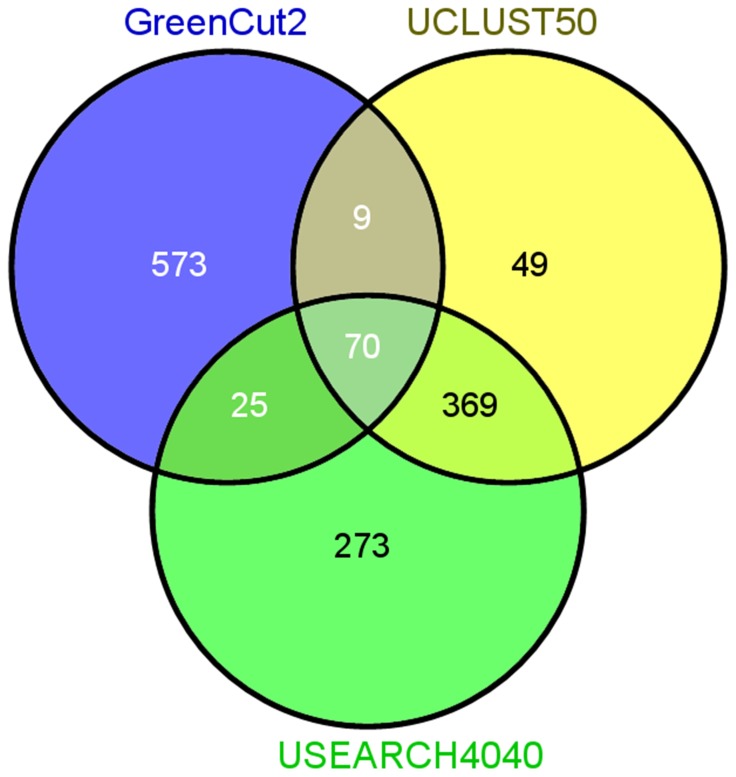
Comparison of conserved plastid-targeted protein datasets with GreenCut2. *Arabidopsis thaliana* loci associated with conserved proteins within the plastid-targeted proteomes identified in this study were compared with those of GreenCut2. A total of 70 sequences were found to be conserved between the three datasets.

While it may be expected that plastid-targeted proteins would be highly conserved in this 7-species analysis, a previous study demonstrated a striking lack of similarity between the plastid proteomes of *Arabidopsis* and *Oryza sativa* [20]. In this study, a predicted *Arabidopsis* chloroplast proteome containing 2,100 proteins shared only 900 with the 4,800 plastid proteins in *Oryza sativa*. These 900 proteins were largely involved in transcription, energy, and metabolism. It would not be surprising to see this shared set of proteins shrink substantially as the number of species compared increases. A large focus has been put on the identification of proteins found only in photosynthetic organisms termed the GreenCut [64]. The first draft of this protein set contained 349 proteins conserved in photosynthetic eukaryotes, and absent in non-photosynthetic organisms. This was later updated generating GreenCut2 with 597 conserved proteins [56]. The comparison of the conserved plastid-targeted protein identified in this study by UCLUST50 and USEARCH40/40 methods reveals that there is only a minor overlap with GreenCut2 (Figure 6). Additionally, clusters do not consist of members from either a single species, or all seven. Instead, there are a significant number of clusters in our analysis with members from various combinations of species. These clusters could be helpful in the identification of proteins involved in plastidial pathways or traits conserved within a set of species. The comparison of more closely related angiosperms may in fact yield more similar plastid proteomes, while including more distantly related angiosperms likely reduces this similarity. It is worth noting that these differences could potentially occur due to the loss or gain of chloroplast transit peptides, rearrangement of protein domains or gene duplication, to name a few plausible mechanisms.

Differences in the outcome of comparative analysis projects can be attributed to the utilization of alignment or clustering methods. Previous studies in microbial comparative proteomic and genomic studies have utilized an alignments of 50% identity and 50% coverage to predictively resolve paralogs from orthologs [65], [66] and 90% nucleic acid identity in algae (Bayer et al., 2012). A previous study which compared plastid proteomes between Arabidopsis and rice utilized BLASTP with e-value cut-off of 10xe^−10^ [20] while that of GreenCut utilized a BLASTP mutual best hit between *Chalydomonas* and Arabidopsis and human to identify paralogs, co-orthologs, and orthologs of *Chlamydomonas* [64]. GreenCut2 further expanded upon these parameters again utilizing mutual best hit analysis with BLASTP cut-off of 10xe^−10^ to identify orthologs and included sequences with over 50% amino acid identity as in-paralogs [56]. For this study, a predicted plastid-targeted protein was considered “unique” to a species if a global USEARCH alignment at 40% identity over 40% of the query sequence matched no sequences from the other 6 species. These parameters were chosen instead of 50% identity and 50% coverage as more matches were identified with a higher confidence with paralogous proteins removed. UCLUST, based on of global alignments, was then utilized to identify clusters with 50% amino acid identity, presumably including both orthologs and in-paralogs [33]. Of course the functionality of a protein cannot be ascertained through sequence identity alone. This study may not identify genes that have undergone rearrangement yet retain similar gene product functions, or examples of convergent evolution. The assumption in this study is that if such arrangements occur and functions are retained, or divergent plants adapt to create the same function in separate gene sequences, that an appropriate sequence from one of the remaining six other species would retain or have similar sequence identity for a 40/40 alignment to occur. However, the datasets that we present could be further investigated in the future through the use of a BLASTP-based comparison, and with other genomes as they become available.

The unique plastid-targeted proteins within each of the investigated species possess varied levels of functional information. Despite the lack of spatial and temporal transcriptome and proteome expression context, this information has a large referential value for future work. Blast2GO analysis and GO term enrichment analysis provide a glimpse as to what these proteins are likely participating in within the plastids. However, only a few GO terms were found to be enriched, most of which differed between species. This suggests that there are likely not specific classes of proteins which fall into this category of “unique” for the plastid proteomes of each species. As expected, due to substantial research performed on it, a large proportion of the proteins unique to the Arabidopsis plastid proteome (98%) possess associated GO terms (Table 8). However, tomato, which is used in the scientific community to understand and characterize the chloroplast to chromoplast transition, has the lowest percentage of unique proteins without GO terms, at 56.5%. Our analysis reinforces the lack of understanding about plastid biology especially in non-model systems, and the urgent need for further functional characterization of novel biological processes that these organelles harbor.

Plastids play an integral part in plant development, photosynthesis, and several other known biochemical processes. However in fruits their role has remained uncharacterized. Several important biochemical processes for synthesis and storage of pigments, nutraceutical and medically important compounds as well as aromatic compounds are resident in fruit plastids. These components are important both for consumer appeal as well as nutritional value. In addition, various physiological disorders, such as sunscald in apple, are associated with the inability of fruits to adequately quench excessive energy from sunlight. Through furthering our understanding of the plastid function in non-model plant systems and organs such as fruit, novel mechanisms for enhancing photosynthetic efficiency and crop productivity could be discovered.

The results presented in this work indicate that the current state of knowledge regarding plastid biology, mostly derived from model systems, is not comprehensive enough. In each plant species evaluated in this work, plastids are predicted to host a plethora of biological and metabolic processes necessitating subsequent wet-lab validation in non-model systems. New plant genomes are expected to enable the identification of potentially new plastid-targeted proteins that will aid in studying novel roles of plastids in plant development, metabolism and adaptation.

## Conclusions

While previous studies have advocated the integration of multiple protein localization prediction techniques [20] it appears that no significant difference exists between a custom analysis utilizing multiple approaches and TargetP analysis for identifying plastid-targeted proteins in apple. Such results suggest that initial data mining with only TargetP may, in fact, be sufficient, depending upon the application. This is with the caveat that TargetP has an approximate 35% false positive rate in detecting plastid-targeted proteins [58]. As the understanding of protein localization improves, and complete genome sequences from larger number of plants become available, such predictive techniques will likely become a more reliable method to generate draft plastid proteomes.

The TargetP-based analysis indicates that a large subset of a plant’s nuclear-encoded proteome is predicted to be localized to the plastid. However, the proportion of transcripts encoding plastid-targeted proteins varies, compromising 10–20% of the transcriptome depending upon the species investigated. Of the nuclear-encoded plastid proteome, there appears to be a significant subset that is species-specific. Many of these proteins have homology to proteins not predicted to be plastid-targeted in other systems, indicating that it may be common for proteins to gain or lose targeting peptides during evolution. If this is indeed the case, it would be interesting to investigate the evolutionary and mechanistic context of gain or loss of target peptides across species.

Through using two comparative methods, a USEARCH-based approach as well as a semi-global UCLUST-based approach, we displayed that very few plastid-targeted proteins are conserved between the predicted nuclear-encoded plastid proteomes. This value varied based upon the comparative technique and user parameters, but our predictions identify 497 and 737 Arabidopsis proteins which contain predicted plastid-targeted homologs in all other examined angiosperms. GO term enrichment analysis suggests that specific functions are significantly conserved in plastids, namely photosynthesis, many metabolic processes, transport, and cell death. Knowledge about these conserved proteins can be utilized in future studies to better understand and potentially predict those proteins which are plastid-targeted in other non-model systems and additionally identify novel plastid-targeted proteins.

The expression of genes encoding plastid-proteins appears to be very diverse within the fruit developmental continuum with 64 significant expression patterns detected (those containing five or more genes). However, most of the genes investigated can be clustered into nine expression patterns. These expression patterns can be overlapped with important milestones within the development of fruit to find plastid proteins which may be responsible for novel fruiting milestones or processes. While expression data is available for ∼13,000 genes, subsequent developmental transcriptomics, metabolomics, and proteomics investigations are expected to provide a comprehensive understanding of the roles of plastids in apple fruit development.

## Supporting Information

File S1 **Expression data from Janssen et al. for** ***Malus × domestica*** **genes predicted to encode plastid-targeted proteins.** Expression data for each cluster of co-expressed genes with sequence header and expression data in the form of Log2 of expression relative to the lowest value.
(XLSX)

File S2 **GO term information for** ***Malus × domestica*** **plastid-targeted protein clusters.**
(XLSX)

File S3 **Header information for predicted plastid-targeted proteins from the investigated seven species.** Excel file containing identifiers of predicted plastid-targeted protein sequences generated through with TargetP analysis.
(XLSX)

File S4 **Plastid-targeted proteins unique to each examined species with GO term information.** Excel file containing the header information and associated GO terms.
(XLS)

File S5 **Plastid-targeted proteins shared between all species.** Excel file containing the header information for the UCLUST50 analysis separated by species. Information is provided for the 737 *Arabidopsis thaliana* sequences shared in the USEARCH comparative analysis.
(ZIP)

File S6 **Blast2GO annotation file containing header and GO term information for 70 protein sequences present in GreenCut2, UCLUST50, and USEARCH4040 analyses.**
(ZIP)

## References

[1] Kutschera U, Niklas K (2005) Endosymbiosis, cell evolution, and speciation. Theory Biosci 124: 1–24. 17046345 10.1016/j.thbio.2005.04.001

[2] Mettal U, Boland W, Beyer P, Kleinig H (1988) Biosynthesis of monoterpene hydrocarbons by isolated chromoplasts from daffodil flowers. Eur J Biochem 170: 613–616. 3338457 10.1111/j.1432-1033.1988.tb13741.x

[3] Kiss JZ, Hertel R, Sack FD (1989) Amyloplasts are necessary for full gravitropic sensitivity in roots of *Arabidopsis thaliana* . Planta 177: 198–206. 11539759

[4] Fischer K, Weber A (2002) Transport of carbon in non-green plastids. Trends Plant Sci 7: 345–351. 12167329 10.1016/s1360-1385(02)02291-4

[5] Martin W, Herrmann RG (1998) Gene transfer from organelles to the nucleus: how much, what happens, and Why? Plant Physiol 118: 9–17. 9733521 10.1104/pp.118.1.9 PMC1539188

[6] Rochaix JD (1997) Chloroplast reverse genetics: new insights into the function of plastid genes. Trends Plant Sci 2: 419–425.

[7] Martin W, Rujan T, Richly E, Hansen A, Cornelsen S, et al (2002) Evolutionary analysis of Arabidopsis, cyanobacterial, and chloroplast genomes reveals plastid phylogeny and thousands of cyanobacterial genes in the nucleus. Proc Natl Acad Sci U S A 99: 12246–12251. 12218172 10.1073/pnas.182432999 PMC129430

[8] Jarvis P (2008) Targeting of nucleus-encoded proteins to chloroplasts in plants. New Phytol 179: 257–285. 19086173 10.1111/j.1469-8137.2008.02452.x

[9] Keegstra K, Olsen LJ, Theg SM (1989) Chloroplastic precursors and their transport across the envelope membranes. Annu Rev Plant Physiol Plant Mol Biol 40: 471–501.

[10] Vonheijne G, Steppuhn J, Herrmann RG (1989) Domain-structure of mitochondrial and chloroplast targeting peptides. Eur J Biochem 180: 535–545. 2653818 10.1111/j.1432-1033.1989.tb14679.x

[11] Zhang XP, Glaser E (2002) Interaction of plant mitochondrial and chloroplast signal peptides with the Hsp70 molecular chaperone. Trends Plant Sci 7: 14–21. 11804822 10.1016/s1360-1385(01)02180-x

[12] Chen MH, Huang LF, Li HM, Chen YR, Yu SM (2004) Signal peptide-dependent targeting of a rice a-amylase and cargo proteins to plastids and extracellular compartments of plant cells. Plant Physiol 135: 1367–1377. 15235120 10.1104/pp.104.042184 PMC519054

[13] Miras S, Salvi D, Ferro M, Grunwald D, Garin J, et al (2002) Non-canonical transit peptide for import into the chloroplast. J Biol Chem 277: 47770–47778. 12368288 10.1074/jbc.M207477200

[14] Schemenewitz A, Pollmann S, Reinbothe C, Reinbothe S (2007) A substrate-independent, 14: 3: 3 protein-mediated plastid import pathway of NADPH: protochlorophyllide oxidoreductase A. Proc Natl Acad Sci U S A. 104: 8538–8543. 10.1073/pnas.0702058104 PMC1895985 17483469

[15] Schein AI, Kissinger JC, Ungar LH (2001) Chloroplast transit peptide prediction: a peek inside the black box. Nucleic Acids Res 29. 10.1093/nar/29.16.e82 PMC55866 11504890

[16] Bannai H, Tamada Y, Maruyama O, Nakai K, Miyano S (2002) Extensive feature detection of N-terminal protein sorting signals. Bioinformatics 18: 298–305. 11847077 10.1093/bioinformatics/18.2.298

[17] Emanuelsson O, Brunak S, von Heijne G, Nielsen H (2007) Locating proteins in the cell using TargetP, SignalP and related tools. Nat Protoc 2: 953–971. 17446895 10.1038/nprot.2007.131

[18] Emanuelsson O, Nielsen H, Brunak S, von Heijne G (2000) Predicting subcellular localization of proteins based on their N-terminal amino acid sequence. J Mol Biol 300: 1005–1016. 10891285 10.1006/jmbi.2000.3903

[19] Small I, Peeters N, Legeai F, Lurin C (2004) Predotar: A tool for rapidly screening proteomes for N-terminal targeting sequences. Proteomics 4: 1581–1590. 15174128 10.1002/pmic.200300776

[20] Richly E, Leister D (2004) An improved prediction of chloroplast proteins reveals diversities and commonalities in the chloroplast proteomes of Arabidopsis and rice. Gene 329: 11–16. 15033524 10.1016/j.gene.2004.01.008

[21] Verde I, Abbott A, Scalabrin S, Jung S, Shu S, et al (2013) The high-quality draft genome of peach (*Prunus persica*) identifies unique patterns of genetic diversity, domestication and genome evolution. Nat Genet 45: 487–494. 23525075 10.1038/ng.2586

[22] Jaillon O, Aury JM, Noel B, Policriti A, Clepet C, et al (2007) The grapevine genome sequence suggests ancestral hexaploidization in major angiosperm phyla. Nature 449: 463–U465. 17721507 10.1038/nature06148

[23] Sato S, Tabata S, Hirakawa H, Asamizu E, Shirasawa K, et al (2012) The tomato genome sequence provides insights into fleshy fruit evolution. Nature 485: 635–641. 22660326 10.1038/nature11119 PMC3378239

[24] Shulaev V, Sargent DJ, Crowhurst RN, Mockler TC, Folkerts O, et al (2011) The genome of woodland strawberry (*Fragaria vesca*). Nat Genet 43: 109–U151. 21186353 10.1038/ng.740 PMC3326587

[25] Tuskan GA, DiFazio S, Jansson S, Bohlmann J, Grigoriev I, et al (2006) The genome of black cottonwood, *Populus trichocarpa* (Torr. & Gray). Science 313: 1596–1604. 16973872 10.1126/science.1128691

[26] Velasco R, Zharkikh A, Affourtit J, Dhingra A, Cestaro A, et al. (2010) The genome of the domesticated apple (*Malus × domestica* Borkh.). Nat Genet 42: 833-+. 10.1038/ng.654 20802477

[27] Kreuz K, Kleinig H (1984) Synthesis of prenyl lipids in cells of spinach leaf – compartmentation of enzymes for formation of isopentenyl diphosphate. Eur J Biochem 141: 531–535. 6086332 10.1111/j.1432-1033.1984.tb08225.x

[28] Camara B, Hugueney P, Bouvier F, Kuntz M, Moneger R (1995) Biochemistry and molecular biology of chromoplast development. Int Rev Cytol 163: 175–247. 8522420 10.1016/s0074-7696(08)62211-1

[29] Boyer J, Liu R (2004) Apple phytochemicals and their health benefits. Nutr J 3. 10.1186/1475-2891-3-5 PMC442131 15140261

[30] Liu YS, Roof S, Ye ZB, Barry C, van Tuinen A, et al (2004) Manipulation of light signal transduction as a means of modifying fruit nutritional quality in tomato. Proc Natl Acad Sci U S A 101: 9897–9902. 15178762 10.1073/pnas.0400935101 PMC470770

[31] Robinson N, Hewitt J, Bennett A (1988) Sink metabolism in tomato fruit. Plant Physiol 87. 10.1104/pp.87.3.727 PMC1054828 16666215

[32] Phan CT (1973) Chloroplasts of the peel and the internal tissues of apple-fruits. Experientia 29: 1555–1557.

[33] Edgar RC (2010) Search and clustering orders of magnitude faster than BLAST. Bioinformatics 26: 2460–2461. 20709691 10.1093/bioinformatics/btq461

[34] Hunter S, Apweiler R, Attwood TK, Bairoch A, Bateman A, et al (2009) InterPro: the integrative protein signature database. Nucleic Acids Res 37: D211–D215. 18940856 10.1093/nar/gkn785 PMC2686546

[35] Krogh A, Larsson B, von Heijne G, Sonnhammer ELL (2001) Predicting transmembrane protein topology with a hidden Markov model: Application to complete genomes. J Mol Biol 305: 567–580. 11152613 10.1006/jmbi.2000.4315

[36] Ostlund G, Schmitt T, Forslund K, Kostler T, Messina DN, et al (2010) InParanoid 7: new algorithms and tools for eukaryotic orthology analysis. Nucleic Acids Res 38: D196–D203. 19892828 10.1093/nar/gkp931 PMC2808972

[37] Youens-Clark K, Buckler E, Casstevens T, Chen C, DeClerck G, et al (2011) Gramene database in 2010: updates and extensions. Nucleic Acids Res 39: D1085–D1094. 21076153 10.1093/nar/gkq1148 PMC3013721

[38] Flicek P, Amode MR, Barrell D, Beal K, Brent S, et al (2011) Ensembl 2011. Nucleic Acids Res 39: D800–D806. 21045057 10.1093/nar/gkq1064 PMC3013672

[39] Kinsella RJ, Kahari A, Haider S, Zamora J, Proctor G, et al.. (2011) Ensembl BioMarts: a hub for data retrieval across taxonomic space. Database. 10.1093/database/bar030 PMC3170168 21785142

[40] Lu Y, Savage LJ, Larson MD, Wilkerson CG, Last RL (2011) Chloroplast 2010: a database for large-scale phenotypic screening of Arabidopsis mutants. Plant Physiol 155: 1589–1600. 21224340 10.1104/pp.110.170118 PMC3091111

[41] Lamesch P, Berardini TZ, Li DH, Swarbreck D, Wilks C, et al (2012) The Arabidopsis Information Resource (TAIR): improved gene annotation and new tools. Nucleic Acids Res 40: D1202–D1210. 22140109 10.1093/nar/gkr1090 PMC3245047

[42] Conesa A, Gotz S, Garcia-Gomez JM, Terol J, Talon M, et al (2005) Blast2GO: a universal tool for annotation, visualization and analysis in functional genomics research. Bioinformatics 21: 3674–3676. 16081474 10.1093/bioinformatics/bti610

[43] Gotz S, Garcia-Gomez JM, Terol J, Williams TD, Nagaraj SH, et al (2008) High-throughput functional annotation and data mining with the Blast2GO suite. Nucleic Acids Res 36: 3420–3435. 18445632 10.1093/nar/gkn176 PMC2425479

[44] Quevillon E, Silventoinen V, Pillai S, Harte N, Mulder N, et al (2005) InterProScan: protein domains identifier. Nucleic Acids Res 33: W116–W120. 15980438 10.1093/nar/gki442 PMC1160203

[45] Myhre S, Tveit H, Mollestad T, Laegreid A (2006) Additional Gene Ontology structure for improved biological reasoning. Bioinformatics 22: 2020–2027. 16787968 10.1093/bioinformatics/btl334

[46] Kanehisa M (2002) The KEGG database. *In silico* simulation of biological processes 247: 91–103.

[47] Kanehisa M, Goto S, Sato Y, Furumichi M, Tanabe M (2012) KEGG for integration and interpretation of large-scale molecular data sets. Nucleic Acids Res 40: D109–D114. 22080510 10.1093/nar/gkr988 PMC3245020

[48] Du Z, Zhou X, Ling Y, Zhang Z, Su Z (2010) agriGO: a GO analysis toolkit for the agricultural community. Nucleic Acids Res 38: W64–70. 20435677 10.1093/nar/gkq310 PMC2896167

[49] Janssen BJ, Thodey K, Schaffer RJ, Alba R, Balakrishnan L, et al.. (2008) Global gene expression analysis of apple fruit development from the floral bud to ripe fruit. BMC Plant Biol 8. 10.1186/1471-2229-8-16 PMC2287172 18279528

[50] Saeed AI, Hagabati NK, Braisted JC, Liang W, Sharov V, et al.. (2006) TM4 microarray software suite. DNA Microarrays, Part B: Databases and Statistics 411: 134-+. 10.1016/S0076-6879(06)11009-5 16939790

[51] Saeed AI, Sharov V, White J, Li J, Liang W, et al.. (2003) TM4: A free, open-source system for microarray data management and analysis. Biotechniques 34: 374-+. 10.2144/03342mt01 12613259

[52] Ben-Dor A, Shamir R, Yakhini Z (1999) Clustering gene expression patterns. J Comput Biol 6: 281–297. 10582567 10.1089/106652799318274

[53] Kaul S, Koo HL, Jenkins J, Rizzo M, Rooney T, et al (2000) Analysis of the genome sequence of the flowering plant *Arabidopsis thaliana* . Nature 408: 796–815. 11130711 10.1038/35048692

[54] Clijsters H (1969) On the photosynthetic activity of developing apple fruits. Qualitas Plantarum et Materiae Vegetabiles 19: 129–140.

[55] Meinke D, Muralla R, Sweeney C, Dickerman A (2008) Identifying essential genes in *Arabidopsis thaliana* . Trends Plant Sci 13: 483–491. 18684657 10.1016/j.tplants.2008.06.003

[56] Karpowicz SJ, Prochnik SE, Grossman AR, Merchant SS (2011) The GreenCut2 resource, a phylogenomically derived inventory of proteins specific to the plant lineage. J Biol Chem 286: 21427–21439. 21515685 10.1074/jbc.M111.233734 PMC3122202

[57] Oliveros JC (2007) VENNY. An interactive tool for comparing lists with Venn Diagrams.

[58] van Wijk KJ, Baginsky S (2011) Plastid proteomics in higher plants: current state and future goals. Plant Physiol 155: 1578–1588. 21350036 10.1104/pp.111.172932 PMC3091083

[59] Barsan C, Sanchez-Bel P, Rombaldi C, Egea I, Rossignol M, et al (2010) Characteristics of the tomato chromoplast revealed by proteomic analysis. J Exp Bot 61: 2413–2431. 20363867 10.1093/jxb/erq070

[60] Kleffmann T, Russenberger D, von Zychlinski A, Christopher W, Sjolander K, et al (2004) The *Arabidopsis thaliana* chloroplast proteome reveals pathway abundance and novel protein functions. Curr Biol 14: 354–362. 15028209 10.1016/j.cub.2004.02.039

[61] Peltier JB, Cai Y, Sun Q, Zabrouskov V, Giacomelli L, et al (2006) The oligomeric stromal proteome of *Arabidopsis thaliana* chloroplasts. Mol Cell Proteomics 5: 114–133. 16207701 10.1074/mcp.M500180-MCP200

[62] Wang YQ, Yang Y, Fei Z, Yuan H, Fish T, et al.. (2013) Proteomic analysis of chromoplasts from six crop species reveals insights into chromoplast function and development. J Exp Bot. 10.1093/jxb/ers375 PMC3580812 23314817

[63] Zybailov B, Rutschow H, Friso G, Rudella A, Emanuelsson O, et al.. (2008) Sorting signals, N-terminal modifications and abundance of the chloroplast proteome. PLoS One 3. 10.1371/journal.pone.0001994 PMC2291561 18431481

[64] Merchant SS, Prochnik SE, Vallon O, Harris EH, Karpowicz SJ, et al (2007) The Chlamydomonas genome reveals the evolution of key animal and plant functions. Science 318: 245–251. 17932292 10.1126/science.1143609 PMC2875087

[65] Friis C, Wassenaar TM, Javed MA, Snipen L, Lagesen K, et al. (2010) Genomic Characterization of *Campylobacter jejuni* Strain M1. PLoS One 5. 10.1371/journal.pone.0012253 PMC2928727 20865039

[66] Lukjancenko O, Ussery DW, Wassenaar TM (2012) Comparative Genomics of Bifidobacterium, Lactobacillus and Related Probiotic Genera. Microb Ecol 63: 651–673. 22031452 10.1007/s00248-011-9948-y PMC3324989

